# Role of PM_2.5_ in the development and progression of COPD and its mechanisms

**DOI:** 10.1186/s12931-019-1081-3

**Published:** 2019-06-13

**Authors:** Junling Zhao, Miao Li, Zhihua Wang, Jinkun Chen, Jianping Zhao, Yongjian Xu, Xiang Wei, Jianmao Wang, Jungang Xie

**Affiliations:** 1 Division of Cardiothoracic and Vascular Surgery, Tongji Hospital, Tongji Medical College, Huazhong University of Science and Technology, Wuhan, 430030 China; 2 Department of Respiratory and Critical Care Medicine, National Clinical Research Center of Respiratory Disease, Tongji Hospital, Tongji Medical College, Huazhong University of Science and Technology, Wuhan, 430030 China; 3 Acadia Junior High School, Winnipeg, MB Canada

**Keywords:** Ambient fine particulate matter, Chronic obstructive pulmonary disease, Lung function, Airway inflammation, Emphysematous lesions

## Abstract

**Background:**

A multitude of epidemiological studies have shown that ambient fine particulate matter 2.5 (diameter < 2.5um; PM_2.5_) was associated with increased morbidity and mortality of chronic obstructive pulmonary disease (COPD). However, the underlying associated mechanisms have not yet been elucidated. We conducted this study to investigate the role of PM_2.5_ in the development of COPD and associated mechanisms.

**Methods:**

We firstly conducted a cross-sectional study in Chinese han population to observe PM_2.5_ effects on COPD morbidity. Then, in vitro, we incubated human bronchial epithelial cells to different concentrations of PM_2.5_ for 24 h. The expression levels of IL-6 and IL-8 were detected by ELISA and the levels of MMPs, TGF-β1, fibronectin and collagen was determined by immunoblotting. In vivo, we subjected C57BL/6 mice to chronic prolonged exposure to PM_2.5_ for 48 weeks to study the influence of PM_2.5_ exposure on lung function, pulmonary structure and inflammation.

**Results:**

We found that the effect of PM_2.5_ on COPD morbidity was associated with its levels and that PM_2.5_ and cigarette smoke could have a synergistic impact on COPD development and progression. Both vitro and vivo studies demonstrated that PM_2.5_ exposure could induce pulmonary inflammation, decrease lung function, and cause emphysematous changes. Furthermore, PM_2.5_ could markedly aggravated cigarette smoke-induced changes.

**Conclusions:**

In short, we found that prolonged chronic exposure to PM_2.5_ resulted in decreased lung function, emphysematous lesions and airway inflammation. Most importantly, long-term PM_2.5_ exposure exacerbateed cigarette smoke-induced changes in COPD.

## Background

Chronic obstructive pulmonary disease, a major public health problem worldwide, is characterized by irreversible and progressive airflow limitation that is associated with chronic and aberrant pulmonary inflammation and pulmonary remodeling induced by abnormal pulmonary response to inhaled noxious particles and gases [1]. In recent years, COPD has been a major cause of global morbidity and mortality. It will become the third leading cause of death and the fifth global burden worldwide by 2020 [2]. Cigarette smoke has been widely recognized as the most important causal factor for COPD for decades, yet only a small part of smokers (15–20%) could eventually develop COPD [3, 4]. Moreover, approximately 25% of COPD patients are non-smokers [5]. Thus, it suggests that other factors also contribute significantly to COPD development and progression. In recent decades, substantial epidemiological evidence indicated that ambient particulate air pollution including PM_2.5_ is a major detrimental risk factor for COPD [6–9]. Moreover, numerous epidemiological studies from large population cohorts demonstrated that PM_2.5_ was not only associated with an enhanced risk of COPD hospitalization, morbidity and mortality [10–12], but also exacerbated and aggravated respiratory function and symptoms of COPD patients, such as shortness of breath, coughing and wheezing [13–15]. Air pollution improvement would substantially provide benefits to COPD patients [16–18]. However, little is known about the mechanisms underlying these effects that PM_2.5_ induces and further aggravates COPD development and progression. Therefore, we conducted this study to explore the influence of chronic prolonged PM_2.5_ exposure on COPD development. Firstly, we conducted a cross-sectional study in Chinese han population to observe PM_2.5_ effects on COPD morbidity and on smokers with COPD. Then, in vitro, we incubated HBEs with different concentrations of PM_2.5_ to investigate the role of PM_2.5_ in COPD development and its mechanisms. Additionally, to further deeply explore the impacts and associated mechanisms of PM_2.5_ on COPD, we conducted chronic prolonged PM_2.5_-exposed animal model for 48 weeks to mimic COPD development over a substantial duration of individual life so as to study the influence of PM_2.5_ exposure on lung function, pulmonary structure and inflammation to further acquire in-depth appreciation of the associated mechanisms of PM_2.5_ on COPD.

## Methods

### Subject

Study subjects were recruited from urban (Wuhan) and rural (Haokou, Qianjiang) settings. The urban cohort including 1372 participants recruited from two communities in Wuhan. The rural cohort including 1843 participants recruited from 15 villages in Haokou, about 200 km away from Wuhan. Study subjects were born in the local area and have resided locally for at least five years. Individuals were diagnosed as COPD on the basis of American Thoracic Society criteria and the Global Initiative for Chronic Obstructive Lung Disease (GOLD) criteria. The inclusion and exclusion criteria of COPD subjects in this study were described in our previous study [19, 20]. Briefly, postbronchodilator forced expiratory volume in one second (FEV1)/forced vital capacity (FVC) < 70%, and FEV1 < 80% predicted was the diagnostic criteria in this study. Subject were excluded from the study if they were diagnosed as asthma and other obstructive syndromes. The existing PM_2.5_ levels per year at the two study sites were obtained from Hubei Provincial Environmental Monitoring Center station (HEWC), which was reported in detail in our previous study [20]. The data were gathered automatically and continuously, 24 h/d and 365 d/y, with no interruption. The monitoring was carried out by the HEMC and authorized by the United States Environmental Protection Agency (EPA).

Written informed consents were obtained from all participants, and the study was approved by the institutional ethics committees of local participating hospitals.

### PM_2.5_ sampling collection and extraction

PM_2.5_ was continuously collected using UAS-310-PM_10–2.5_ and PM_2.5_ high-volume air sampler (MSP, USA) at a flow rate of 300 L /min from ambient air in the campus of Tongji Medical College, Huazhong University of Science and Technology, Wuhan, China on Teflon microporous membranes and stored at − 20 °C in the dark until extracted. The filters containing PM_2.5_ were divided into small pieces of approximately 1 cm^2^ and then immersed in sterile purified water before sonicating for 4 × 30 min in a water bath with an ultrasonic cleaner. After drying by lyophilisation, PM_2.5_ dry powder was stored at − 80 °C until use.

### Cell culture and stimulation

HBEs were purchased from ATCC (Manassas, VA, USA). Cells were cultured in 1640 RPMI medium (HyClone, USA) containing 100 units/mL penicillin and 100 μg/mL streptomycin (KeyGEN, Nanjing, China) and supplemented with 10% fetal bovine serum (Gibco, USA). The cells were maintained in a humidified atmosphere of 5% CO_2_ at 37 °C. Before stimulation, cells were cultured in serum-free medium for 24 h to arrest and synchronize the cell growth. Then they were treated with different final concentrations of freshly dispersed PM_2.5_ preparations with or without cigarette smoke extract (CSE, 25 μg/ml) (Murty Pharmaceuticals, Inc., Lexington, KY, USA) for 24 h. After 24 h stimulation, cell culture supernatants were collected, centrifuged and stored at − 80 °C for futher analysis.

### Western blot analysis

Total proteins were collected in appropriate ice-cold RIPA lysis buffer for immunoblotting analysis. Anti-MMP9 Ab (Santa Cruz, CA), anti-MMP12 Ab (Abcam, Cambridge, UK), collagen I (Abcam, Cambridge, UK), collagen III (Abcam, Cambridge, UK), anti-TGFβ1 Ab (Proteintech Group, China), fibronectin (Proteintech Group, China) and anti-β-actin (Proteintech Group, China) were used. Image J was used to quantify the intensity of the protein band, which was normalized to β-actin in the analyses.

### Animals

Six-week-old male C57BL/6 mice were obtained from Hubei Provincial Laboratory Animal Public Service Center, Wuhan, Hubei Province, China. They were equilibrated for 2 weeks before exposure experiment. The mice were fed a normal diet and housed in cages at the Experimental Animal Centre, Tongji Medical College, Huazhong University of Science and Technology. All animal experimental procedures were authorized by Huazhong University Animal Experiment Ethics Committee and executed in accordance with institutional regulations for ethical animal use.

### Animal experimental procedures

C57BL/6 mice were randomly divided into four groups (*n* = 6 per group): control group, CS-exposed group, PM_2.5_-exposed group (PM_2.5_) and PM_2.5_ + CS exposed group (PM_2.5_ + CS). The CS-exposed procedure was carried out as described previously [21]. Briefly, CS-exposed mice were exposed to cigarette smoke produced by 5 cigarettes twice daily, 5 d/wk., using a PAB-S200 Animal Passive Smoking Whole Body Exposure System (BioLab Technology Co. Ltd., Beijing, China) for continuously for 48 weeks. PM_2.5_-exposed mice were placed in a 192 L exposure chamber (0.8 m in length, 0.6 m in width and 0.4 m in height) with three holes of 10 mm in diameter on the top panel of the chamber for fresh air inlet and one hole of 30 mm in diameter on the side panel of the chamber, through which PM_2.5_ aerosols generated by an ultrasonic nebulizer to the exposure chamber. PM_2.5_-exposed C57BL/6 mice, in an awake and unrestrained state, were subjected to concentrated ambient air PM_2.5_ in whole-body inhalation protocol in vivo for continuously for 48 weeks. The PM_2.5-_exposed procedures were referred as described previously [22]. In detail, the ambient daily average PM_2.5_ concentration close to our study site was 101.3μg/m^3^, which was much higher than that listed in the Global Air Quality Guidelines from the World Health Organization (annual average: 10 μg/m^3^). The average PM_2.5_ concentration in the exposure room was 560μg/m^3^, equivalent to 5.7-fold level of ambient daily PM_2.5_. Since the mice were exposed for 6 h/d for 5 d/wk in concentrated PM_2.5_ exposure group, the equivalent PM_2.5_ concentration for mice exposed in the exposure room, after normalization over the exposure study period, was 110 μg/m^3^, which was within the annual average concentrations PM_2.5_. At the same time, mice in PM_2.5_ + CS exposed group was exposed to cigarette smoke followed by PM_2.5_ as the methods described above. Simultaneously, the controls were exposed to filtered air.

### Lung function measurement

Lung function was measured by using AniRes 2005 lung function system (Bestlab, Beijing, China) as previously described [23]. In detail, the mice were anaesthetized with 1% pentobarbital sodium by means of intraperitoneaI injection and subsequently a tracheal cannula was inserted. Then, the mice were immediately placed supine in a sealed whole-body plethysmograph and connected to a computer-controlled ventilator through the tracheal cannula. All animals were ventilated mechanically at respiratory rates of 90 breaths/min with a tidal volume of 5 ml/kg and at expiration/inspiration time ratio of 1.5:1.0 with a computer-controlled and assisted small animal ventilator. Forced expiratory vital capacity (FVC), Forced expiratory volume in 0.1 s (FEV_0.1_) and FEV_0.1_/ FVC were measured after the mouse was acclimatized for at least 30s and all parameters were stable.

### Pulmonary morphometric and pathology assessment

After pulmonary function measurements, the chests of the mice were immediately opened, and the tracheas were intubated with a tracheal cannula. Before performing bronchoalveolar lavage (BAL), we ligated the left main bronchus to avoid any impacts of operations on pulmonary morphometric and pathology assessment. Then, the right lung was instilled with 0.8 ml of PBS X 3 via PE-90 tubing to acquire the BAL fluid. The first BAL fluid was centrifuged to remove cells, and the cell-free supernatants were collected and stored at − 80 °C for ELISA analysis. Afterwards, the right lung was excised and stored at − 80 °C for further analysis. The left lung was not lavaged, but in contrast was inflated and fixated with 4% paraformaldehyde through the tracheal cannula, immersed in fresh 4% paraformaldehyde after excision, and then embedded in paraffin. Lung tissue was stained with hematoxylin and eosin (H&E) and analyzed by light microscopy for histology and morphometric examination. We determined enlargement of alveolar spaces by measuring mean linear intercept (MLI) as described previously [24]. Additionally, collagen deposition was determined by using Sirius Red according to the manufacturer’s instructions. Peribronchial collagen deposition in lung tissue of mice was assessed as previously described [25]. In detail, the length of the basement membrane (Pbm) was manually marked on the digital representation of the airway. For the quantification of collagen deposition, the software (KS400; Zeiss) was used to determine the area in the airway wall covered by collagen and its value was counted. The area of collagen deposition was normalized to Pbm. Three lung sections per animal in every experimental group were examined.

### Immunohistochemistry

The lung sections acquired from formalin-fixed, paraffin-embedded left lung lobes were stained with anti-TGF-β1-Ab (Abcam, Cambridge, UK), anti- MMP9-Ab (Santa Cruz, CA), anti-MMP12-Ab (Proteintech, China) and anti-fibronectin Ab (Proteintech, China), respectively, according to the manufacturers’ instructions. As described previously [23], the mean integral OD of MMP9, MMP12, TGF-β1 and fibronectin protein staining in the bronchiolar epithelium was detected. The mean integral OD of protein staining was the ratio of the integral OD of protein staining-positive epithelium to the area of corresponding bronchial epithelium. The same parameters was used for all of the images.

### Enzyme linked immunosorbent assay (ELISA)

The *cytokine and chemokine* protein levels in the BALF and cell culture supernatants were determined using ELISA. Human IL-6 and IL-8 in cell culture supernatants and mouse IL-6 and KC (mouse IL-8) in BAL were separately determined by ELISA kits from R&D Systems Inc. (Minneapolis, MN, USA) according to the manufacturer’s instructions.

### Statistical analysis

All statistical analyses were carried out by using IBM SPSS 19.0 and GraphPad Prism5.0. Categorical variables were expressed as counts and percentages, and chi-square tests were used to compare these variables. Continuous variables were reported as mean ± standard deviation (SD). Normally distributed variables were assessed by using Student’s t test for two groups or one-way ANOVA with Newman-Keuls post hoc test for multiple comparisons and abnormal distribution variables were evaluated by Mann–Whitney U-test. Multiple logistic regression analysis were used to find out potential confounders associated with COPD onset. *P* < 0.05 was considered as statistical significance.

## Results

### Subject characteristics

The baseline characteristics of the cohorts are summarized in Table 1. Briefly, the urban cohort included 1372 participants, and the rural cohort included 1843 participants. Of these subjects, 73.8% were male in the urban cohort and 79.2% were male in the rural cohort. Among the urban participants, 86.1% were over 40 years old, and 87.7% were over 40 years old in the rural cohort. There were no statistically significant differences in age distribution between the two cohorts. The percentage of those who never-smoke in the urban cohort was markedly higher than that of the rural population (urban, 74.3% vs 40.5%, rural). Smokers in the urban cohort was significantly lower than that in the rural cohort (*P* < 0.05). Moreover, 18.9% of the urban cohort had a smoking history above 10 pack-years, which was significantly lower than the rural cohort. The subjects in the study were all born in their current city/area and lived there for more than five years.

**Table 1:** Baseline characteristics of the study participants

	Urban (Wuhan)	Rural (Haokou)	*P*-value
No. subjects	1372	1843	
<40y, No. subjects/Total(%)	191/1372 (13.9)	226/1843 (12.3)	
≥40y, No. subjects/Total(%)	1181/1372 (86.1)	1617/1843 (87.7)	0.17
Gender			
Male, n/T(%)	1013/1372 (73.8)	1294/1843 (79.2)	
Female, n/T(%)	359/1372 (26.2)	549/1843 (20.8)	0.02
Smoking status			
Smoking(>0), n/T (%)	352/1372 (25.7)	1097/1843 (59.5)	
Never smoking (=0), n/T (%)	1020/1372 (74.3)	746/1843 (40.5)	< 0.001
Smoking(≥10), n/T (%)	259/1372 (18.9)	1000/1843 (54.3)	
Smoking(<10), n/T (%)	1113/1372 (81.1)	843/1843 (45.7)	< 0.001
Smoking amount, pack-year (mean ± SD)			
Total	6.2 ± 15.1	20.8 ± 27.6	< 0.001
Smoking(>0)	24.3 ± 21.3	35.1 ± 28.1	< 0.001
Never smoking(=0)	0	0	Not Applicable
Smoking(≥10)	31.4 ± 20.6	38.0 ± 27.7	< 0.001
Smoking(<10)	0.4 ± 1.5	0.5 ± 1.8	0.18
Lung function			
Total, n/T (%)	1372/1372 (100)	1843/1843 (100)	
FVC, ml (mean ± SD)	3.37 ± 0.83	3.40 ± 0.82	0.31
FEV1, ml (mean ± SD)	2.73 ± 0.71	2.68 ± 0.76	0.057
FEV1/FVC, % (mean ± SD)	81.22 ± 8.10	78.31 ± 10.45	< 0.001
Smoking(>0), n/T (%)	352/1372 (25.7)	1097/1843 (59.5)	
FVC, ml (mean ± SD)	3.97 ± 0.73	3.51 ± 0.84	< 0.001
FEV1, ml (mean ± SD)	3.08 ± 0.69	2.68 ± 0.81	< 0.001
FEV1/FVC, % (mean ± SD)	77.55 ± 9.80	75.33 ± 11.18	< 0.001
Never smoking (=0), n/T (%)	1020/1372 (74.3)	746/1843 (40.5)	
FVC, ml (mean ± SD)	3.17 ± 0.76	3.23 ± 0.77	0.1
FEV1, ml (mean ± SD)	2.61 ± 0.68	2.68 ± 0.68	0.03
FEV1/FVC, % (mean ± SD)	82.49 ± 6.99	82.71 ± 7.32	0.52
Smoking(≥10), n/T (%)	259/1372 (18.9)	1000/1843 (54.3)	
FVC, ml (mean ± SD)	3.89 ± 0.69	3.48 ± 0.83	< 0.001
FEV1, ml (mean ± SD)	2.95 ± 0.63	2.64 ± 0.80	< 0.001
FEV1/FVC, % (mean ± SD)	75.74 ± 10.18	74.85 ± 11.13	0.24
Smoking(<10), n/T (%)	1113/1372 (81.1)	843/1843 (45.7)	
FVC, ml (mean ± SD)	3.25 ± 0.81	3.30 ± 0.81	0.18
FEV1, ml (mean ± SD)	2.68 ± 0.72	2.73 ± 0.70	0.12
FEV1/FVC, % (mean ± SD)	82.50 ± 6.94	82.42 ± 7.80	0.81
COPD morbidity			
Total, n/T (%)	92/1372 (6.7)	229/1834 (12.5)	< 0.001
Smoking(>0), n/T (%)	52/352 (14.8)	208/1097 (19.0)	0.075
Never smoking (=0), n/T (%)	40/1020 (3.9)	21/746 (2.8)	0.21
Smoking(≥10), n/T (%)	49/259 (18.9)	203/1000 (20.3)	0.62
Smoking(<10), n/T (%) mm	43/1113 (3.9)	26/843 (3.1)	0.36

### The association between PM_2.5_ and the incidence of COPD

**Fig. 1:**
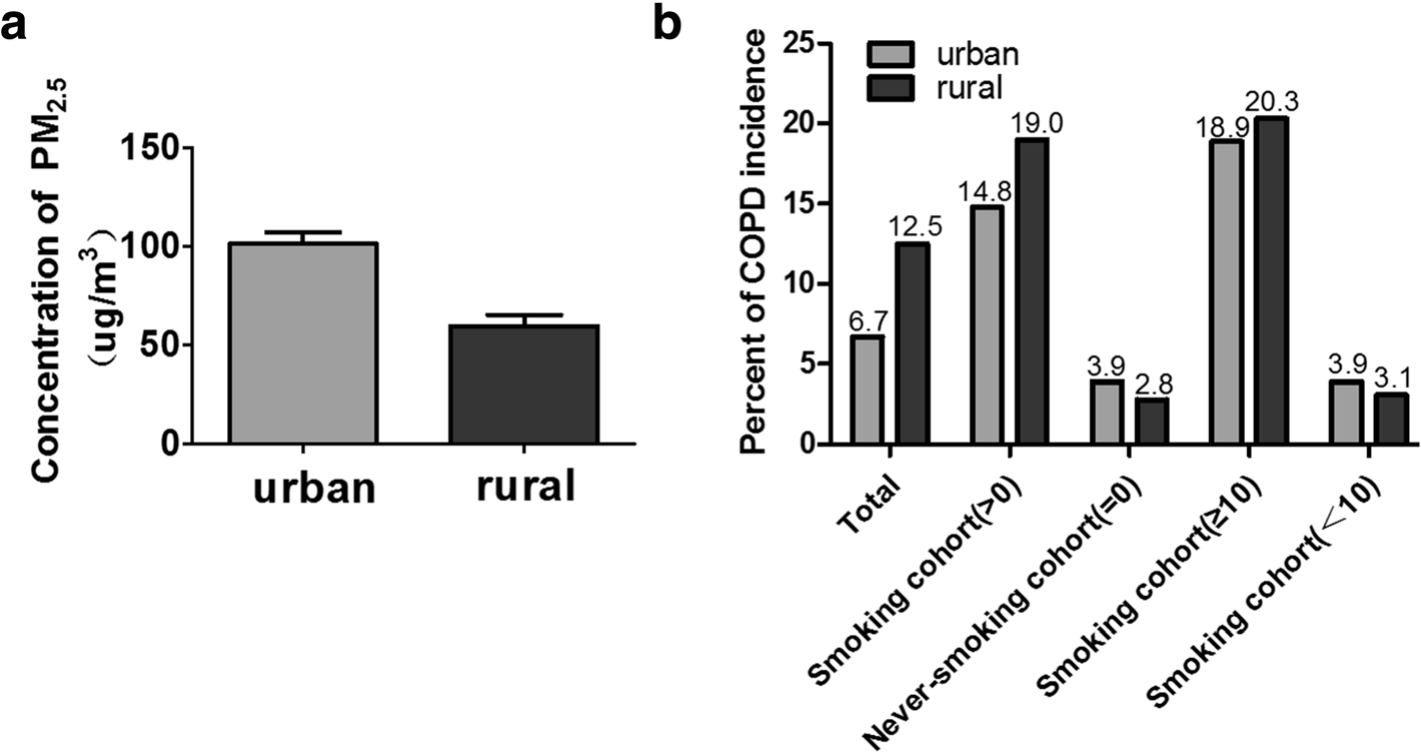
The association of PM_2.5_ with COPD incidence in the population study. **a** The levels of PM_2.5_ in urban and rural settings; **b** COPD incidence among different groups based on smoking-pack-years

As shown in Fig. 1a, mean PM_2.5_ concentration in the urban was obviously higher than the rural (101.3 μg/ml vs 59.4 μg/ml). Meanwhile, in non-smoking cohorts, COPD incidence in the urban was higher than that of the rural, while not statistically significant (Fig. 1b). Interestingly, compared to never-smoking cohorts, COPD incidence in cigarette smoking cohorts increased largely in both areas. Multiple logistic regression analysis were used to find out potential confounders (including age, gender, smoking status as well as PM_2.5_ explore) associated with COPD onset. The results was that no statistical correlation of the other confounders and COPD, except smoking and PM_2.5_. Taken together, the results indicated that the effect of PM_2.5_ on COPD incidence in the population could be associated with its atmospheric concentration. Moreover, PM_2.5_ and cigarette smoking could have a synergistic effect on COPD development and progression.

### PM_2.5_ augmented inflammatory cytokines and growth factors expression in HBEs

**Fig. 2:**
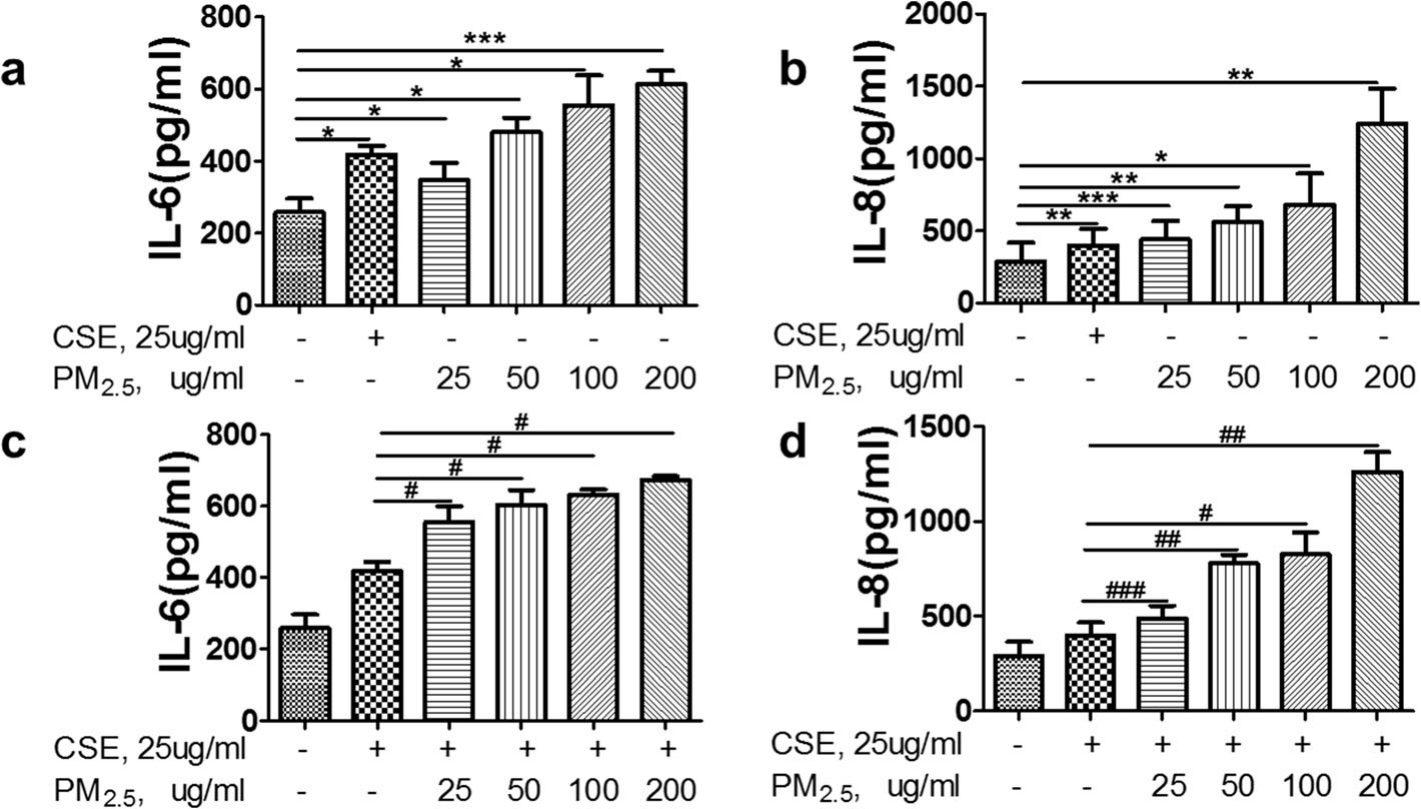
Effects of PM_2.5_ on the cytokines release of IL-6 and IL-8 in HBEs. HBEs were, respectively, incubated with CSE (25 μg/ml) or 25 μg/ml to 200 μg/ml PM_2.5_ for 24 h to examine the levels of pro-inflammatory cytokines by ELISA. **a** IL-6, **b** IL-8. HBEs were co-stimulated with CSE and different concentrations of PM_2.5_ for 24 h to examine the levels of proinflammatory cytokines by ELISA. **c** IL-6, **d** IL-8.The results are mean ± SD of three independent experiments. *P < 0.05, ** *P* < 0.01, *** *P* < 0.001 vs control group; ^#^*P* < 0.05, ^##^ *P* < 0.01, ^###^ *P* < 0.001 vs CSE group

**Fig. 3:**
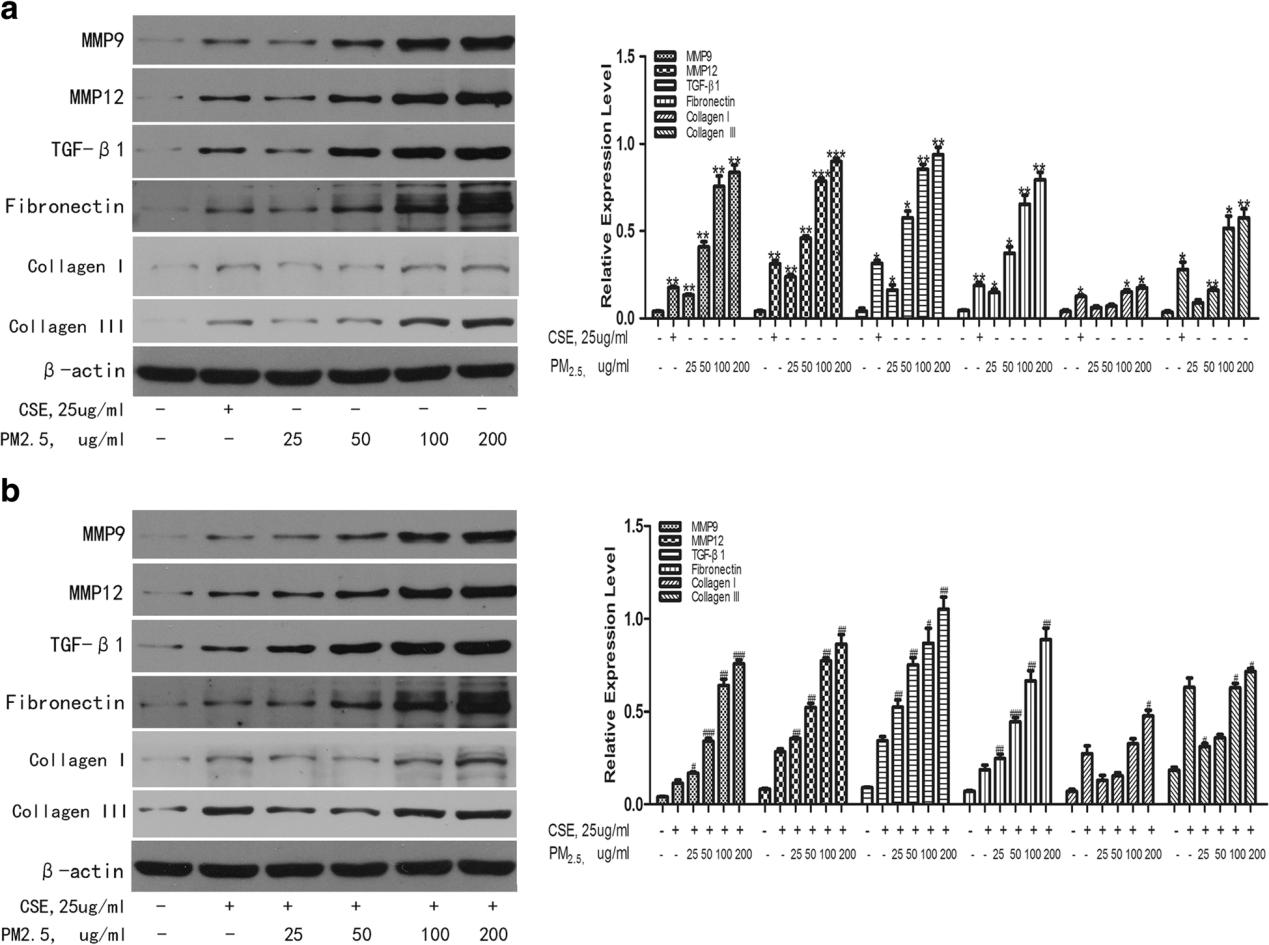
Effects of PM_2.5_ on the protein expression levels of MMPs, TGF-β1, fibronectin and collagen proteins. HBEs were incubated with PM_2.5_ with or without CSE for 24 h. The protein expression levels of MMPs, TGF-β1, fibronectin and collagens were detected by WB. **a** The protein expression of MMPs, TGF-β1, fibronectin and collagen induced by PM_2.5_ /CSE. **b** The protein expression levels of MMPs, TGF-β1, fibronectin and collagens induced by PM_2.5_ + CSE. * *P* < 0.05, ** *P* < 0.01, *** *P* < 0.001 vs control group; ^#^*P* < 0.05, ^##^ *P* < 0.01, ^###^ *P* < 0.001 vs CSE group

Cytokines and chemokines play important roles in the development of COPD [26, 27]. To examine the proinflammatory potential of PM_2.5_, we assessed the production of cytokines by measuring IL-6 and IL-8 levels in cell supernatants. HBEs were incubated with 25 μg/ml to 200 μg/ml PM_2.5_ either with or without CSE for 24 h. Then, IL-6 and IL-8 levels in cell culture supernatants were determined by ELISA. With the lowest concentration of PM_2.5_ (25μg/ml) examined, the levels of IL-6 was increased from a basal level of 258.08 ± 75.91 pg/ml up to 346.78 ± 84.43 pg/ml and the levels of IL-8 from a basal level of 290.36 ± 103.61 pg/ml up to 443.16 ± 104.10 pg/ml (Fig. 2a, b). Moreover, a concentration–response effect were observed in the release of IL-6 and IL-8 in response to PM_2.5_, reaching 613.31 ± 63.72 pg/ml for IL-6 and 1238.99 ± 200.81 pg/ml for IL-8, with the highest concentration of PM_2.5_ (200 μg/ml). Moreover, compared to CSE-exposed group, PM_2.5_ could significantly enhanced the levels of IL-6 production from 418.737 ± 42.74 pg/ml to 555.78 ± 61.89 pg/ml and the levels of IL-8 production from 398.61 ± 94.01 pg/ml to 488.70 ± 92.51 pg/ml at the lowest concentration of PM_2.5_ (25μg/ml) co-stimulated with CSE (Fig. 2c and d). A dose–response effect was also observed by co-stimulation of all four concentrations of PM_2.5_ and CSE, with the level of IL-6 up to 673.68 ± 13.82 pg/ml and IL-8 up to 1260.625 ± 147.00 pg/ml by using the highest concentration of PM_2.5_ (200 μg/ml) together with CSE. Similarly, PM_2.5_ exposure could also obviously upregulate MMP9, MMP12, TGF-β1, fibronectin and collagen protein expression at a concentration-dependent manner and increased CS-induced these proteins expression (Fig. 3a, b). All the above results indicated that PM_2.5_ could induce the enhanced effects of CS-induced inflammatory cytokine secretion and growth factors expression.

### PM_2.5_ exposure impaired pulmonary function in mice

Assessment of the lungs after PM_2.5_/CS exposure for 48 weeks indicated significant lung function decline. As shown in Table 2, ratio of FEV_0.1s_ to forced vital capacity (FEV_0.1s_/FVC) in PM_2.5_ / CS-exposed mice were all less than 70%, while the controls were greater than 70%. Besides obvious decrease of FEV_0.1s_/FVC in PM_2.5_ and CS-exposed group individually, we observed that FEV_0.1s_/FVC was significantly decline in PM_2.5_ + CSE group, implying that PM_2.5_ and CS exposure might synergistically lead to greater damage to pulmonary function than PM_2.5_/CS treatment alone. In addition, the FEV_0.1_ of PM_2.5_+ CS-exposed mice significantly decreased compared to that of any other group, although there were only slight decreases in the FEV_0.1_ in PM_2.5_ /CS-exposed mice, and no statistically significant difference was found in comparison with control mice. Interestingly, PM_2.5_/CS-exposed groups had slightly increased FVC values, while the PM_2.5_ & CS-exposed mice had slightly decreased FVC values.

**Table 2:** Pulmonary function measurement in mice model

Group	FEV_0.1_/FVC	FEV_0.1_ (ml)	FVC (ml)
Control group	75.44 ± 1.64	0.76 ± 0.09	1.04 ± 0.14
CS exposure group	62.73 ± 1.04* ^#^	0.74 ± 0.07^#^	1.19 ± 0.12
PM_2.5_ exposure group	67.59 ± 3.50 ^#^	0.74 ± 0.11^#^	1.09 ± 0.13
CS & PM_2.5_ exposure group	50.35 ± + 4.74*	0.43 ± 0.05*	0.87 ± 0.07

Values are mean ± SD, *n* =6 mice per group; ∗*P <* 0.05 in comparison to control group; ^#^*P <* 0.05 in comparison to PM_2.5_ + CS exposure group

### PM_2.5_ exposure induced pulmonary emphysema changes: airspace enlargement

**Fig. 4:**
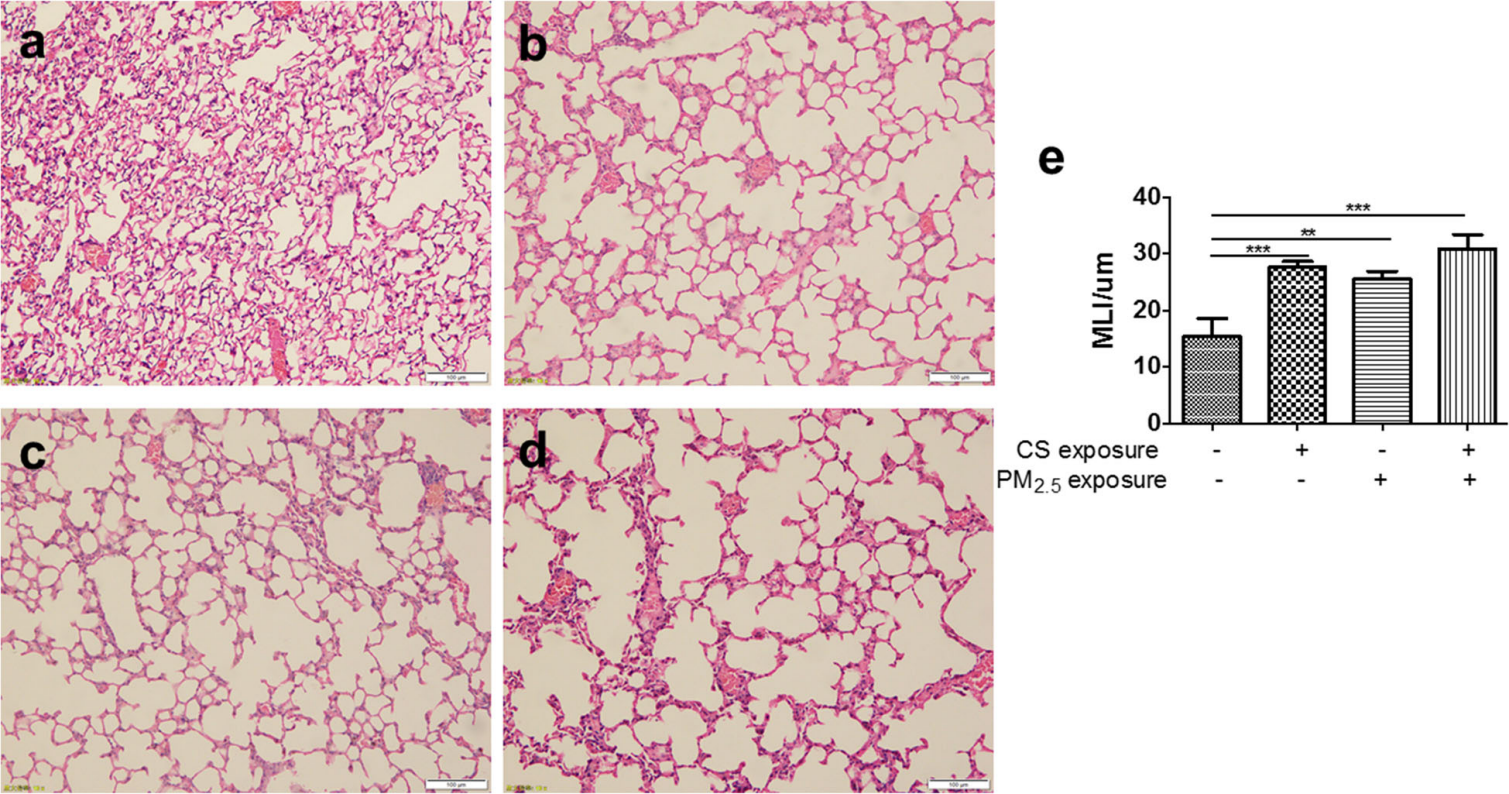
Morphological and pathological changes of lung tissue in mouse model. The representative micrographs of H&E-stained lung tissue of long-term exposure to PM_2.5_ with or without CS for 48 weeks (**a**-**d**) (200x). **a** control group; **b** CS-exposed group; **c** PM_2.5_-exposed group; **d** PM_2.5_ + CS -exposed group. **e** Quantitative morphometric measurements of MLI. ** *P* < 0.01, *** *P* < 0.001 vs control group

Compared to control group, PM_2.5_/CS exposure markedly led to airspace enlargement, respectively, as clearly demonstrated by histological examination of mice lung tissue sections stained with hematoxylin and eosin (HE) in Fig. 4. Remarkably, a clearly stronger enlargement in alveolar spaces was induced by PM_2.5_ + CS exposure in comparison with the other three groups. To further quantify the differences in airspace enlargement between groups, we also measured mean linear intercept (MLI), which has been recognized as a good and accurate indicator for measuring emphysematous changes [28, 29]. An obvious increase in MLI was observed both in chronic PM_2.5_ and CS exposure groups, and a markedly greater airspace enlargement was induced in PM_2.5_ + CS exposure group, confirming emphysematous changes under chronic PM_2.5_ with or without cigarette smoke exposure.

### PM_2.5_ exposure induced pulmonary inflammation and aggravated CS-induced inflammatory changes in mice

**Fig. 5:**
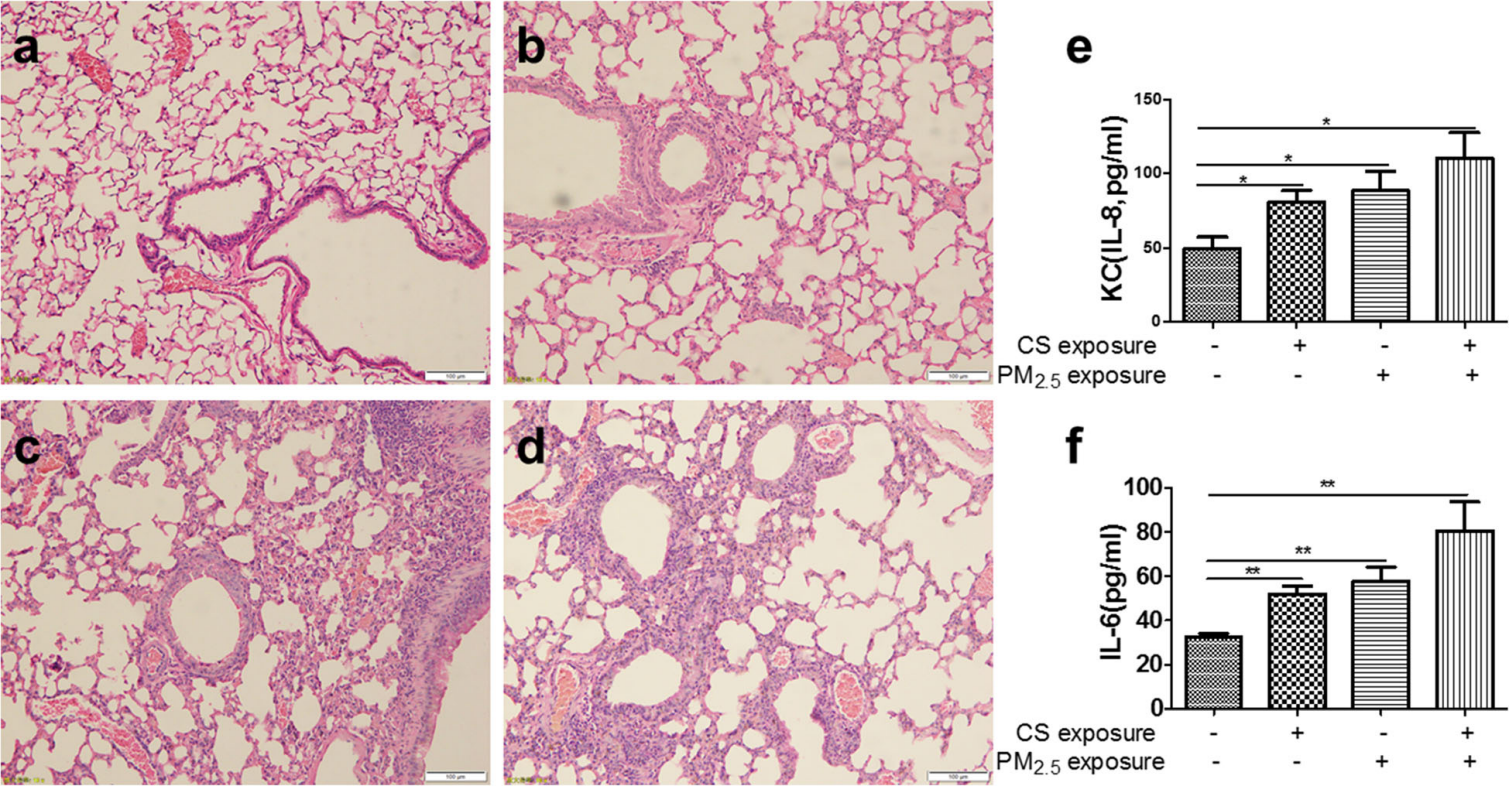
Effect of PM_2.5_ and cigarette smoke exposure on pulmonary inflammation. The representative changes of pulmonary inflammation after long-term exposure to PM_2.5_ with or without CS for 48 weeks (**a**-**d**) (200x). **a** control group; **b** CS-exposed group; **c** PM_2.5_-exposed group; **d** PM_2.5_ + CS -exposed group. The levels of inflammatory cytokines IL-6 and KC (mouse IL-8) in BAL were detected by ELISA; **e** the level of IL-8 in BAL and **f** the level of IL-6 in BAL. Data are presented as mean ± SD (*n* = 6). * *P* < 0.05, ** *P* < 0.01 vs control group

COPD is characterized by pulmonary emphysema and inflammation. To assess pulmonary inflammation, lung histopathology and BALF pro-inflammatory cytokine levels were detected. As shown in Fig. 5, it revealed that there was striking pulmonary inflammation in the peri-bronchial, perivascular and alveolar spaces of the lung in PM_2.5_/CS exposure. Moreover, PM_2.5_ + CS exposure group showed slightly increased inflammation compared to PM_2.5_/CS exposure (Fig. 5a-d). In addition, the levels of pro-inflammatory cytokines IL-6 and KC (IL-8 functional homologs) in the BALF supernatant were clearly elevated separately in PM_2.5_-exposed (IL-6,CON 32.50 pg/ml vs PM_2.5_ 57.89 pg/ml, *p* = 0.0082)(KC,CON 49.65 pg/ml vs PM_2.5_ 72.01 pg/ml, *p* = 0.0305) and CS-exposed group (IL-6,CON 32.50 pg/ml vs CS 52.00 pg/ml, *p* = 0.0029)(KC,CON 49.65 pg/ml vs CS 80.70 pg/ml, *p* = 0.0248) compared to control group. Compared to PM_2.5_/CS-exposed group, PM_2.5_ + CS exposure group showed slightly increased levels of IL-6 and KC, although there was no statistically significant difference.

### PM_2.5_ enhanced MMP9, MMP12 and TGF-β1 protein expression in mice lung

**Fig. 6:**
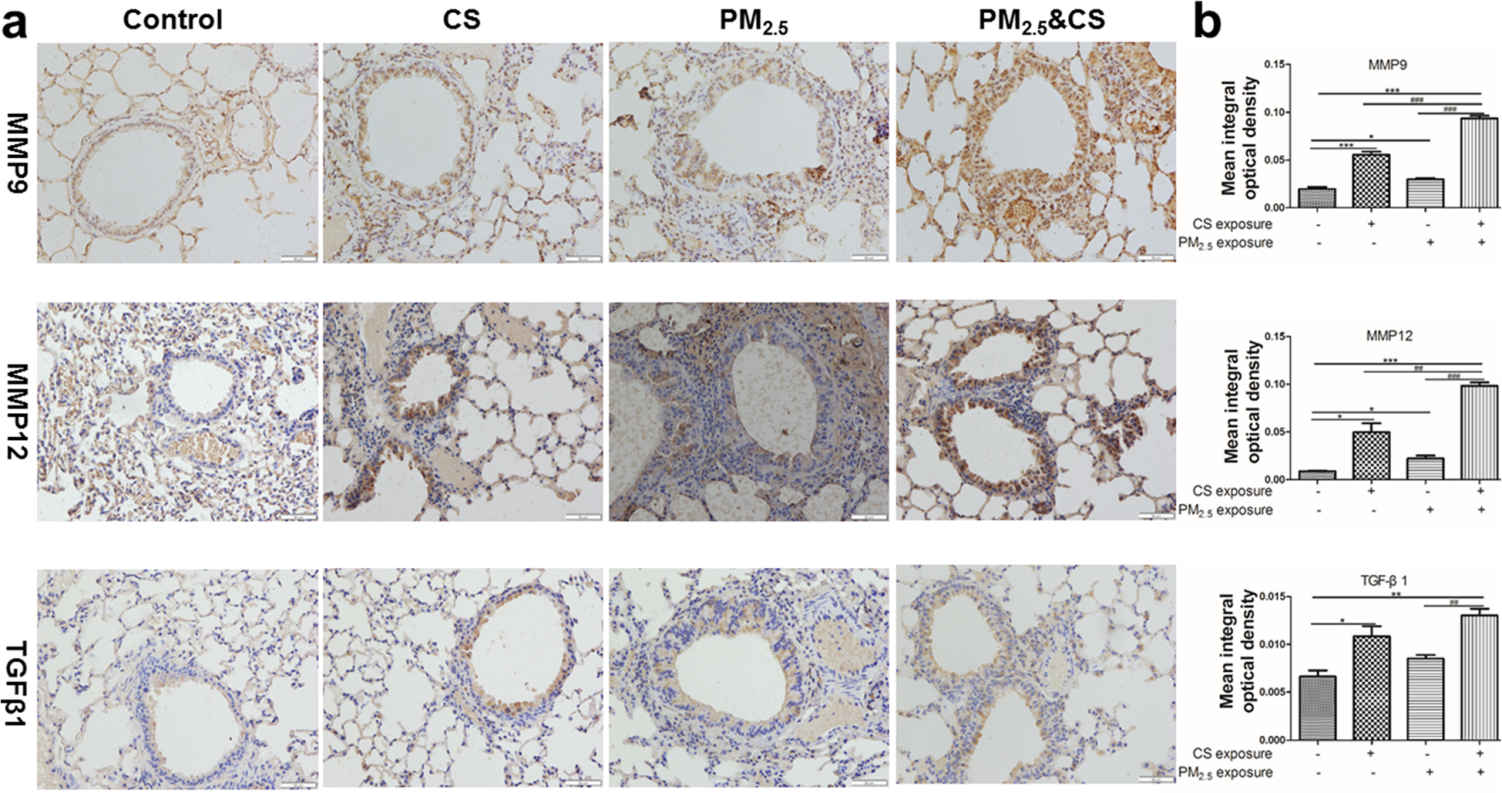
Immunohistochemical staining of MMP9, MMP12, and TGF-β1 in lungs sections induced by PM_2.5_ exposure (400x) (**a**) and semiquantitative assessment of MMP9, MMP12, and TGF-β1 protein expression using Image-Pro Plus (**b**). Data are presented as mean ± SD (*n* = 6). *P < 0.05, ** *P* < 0.01, *** *P* < 0.001 vs control group; ^#^*P* < 0.05, ^##^ *P* < 0.01, ^###^ *P* < 0.001 vs PM_2.5_ + CSE-exposed group

**Fig. 7:**
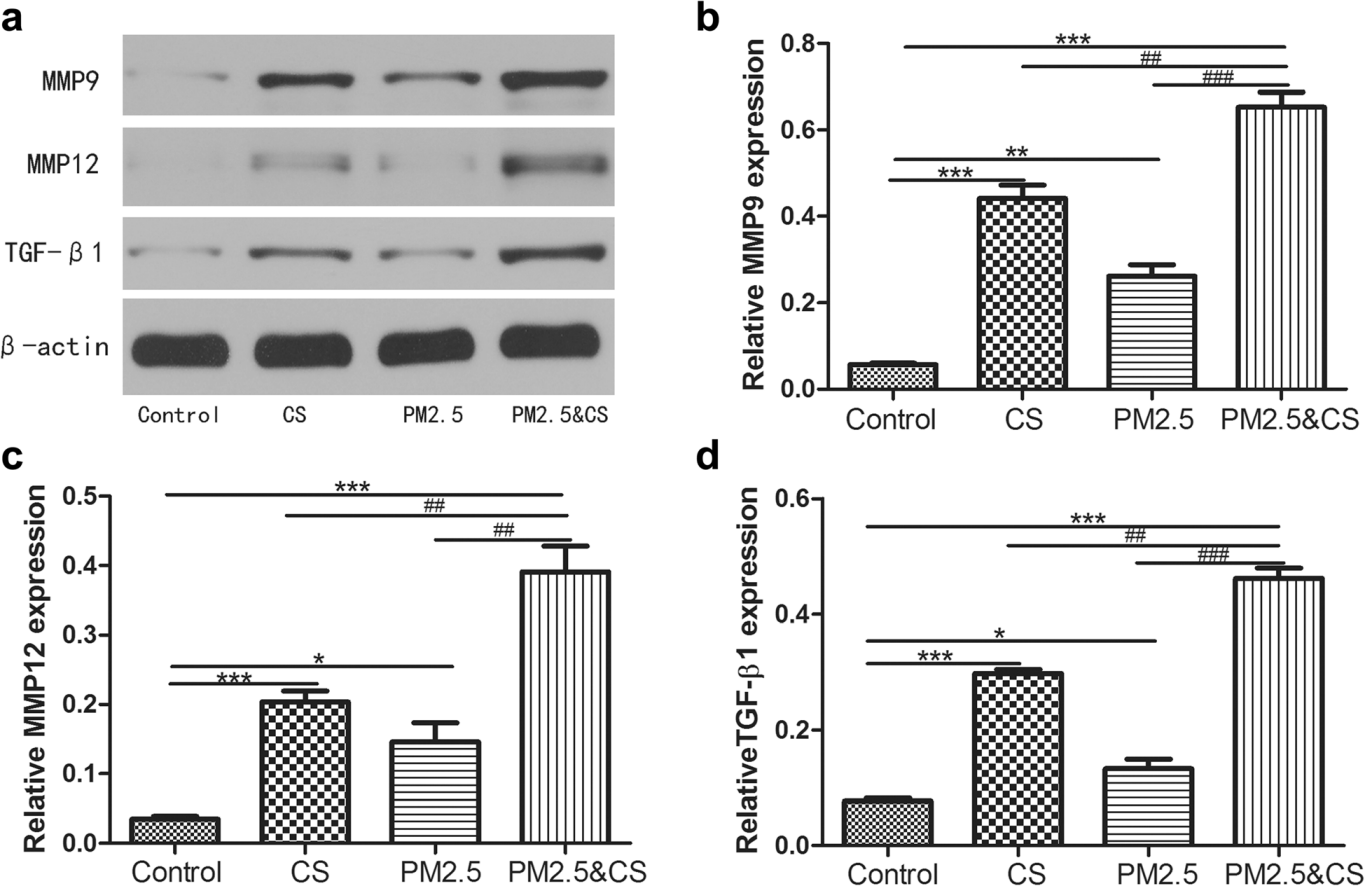
Western blot examination for MMP9, MMP12, and TGF-β1 protein levels expression in lungs sections induced by PM_2.5_/CS exposure. The expressions of MMP9, MMP12, and TGF-β1 were analyzed with western blotting (**a**) and (**b**-**d**) the proteins expression were evaluated. Data are expressed as mean ± SD (*n* = 6). *P < 0.05, ** *P* < 0.01, *** *P* < 0.001 vs control group; ^#^*P* < 0.05, ^##^ *P* < 0.01, ^###^ *P* < 0.001 vs PM_2.5_ + CSE-exposed group

In present study, the effects of PM_2.5_/CS exposure in modulating the protein expression levels of MMP9, MMP12 and TGF-β1 were determined by immune-histochemical analysis and Western blots in mice lung sections (Figs. 6 and 7). Compared with control group, PM_2.5_/CS -exposed group induced significant increases in MMP-9 and MMP12 protein expression evaluation by immunohistochemistry (Fig. 6) and by immunoblot (Fig. 7). Moreover, the protein expression levels of MMP9 and MMP12 in PM_2.5_ + CS group were significantly enhanced relative to any other stimulus group. In contrast, TGF-β1 protein levels in PM_2.5_/CS group were only slightly increased compared with control group. Although TGF-β1 protein expression was significantly increased in PM_2.5_ + CS group compared with that of PM_2.5_ group, there had no significant difference between PM_2.5_ + CS group and PM_2.5_ group.

### Effect of PM_2.5_ and cigarette smoke exposure on airway wall remodeling

**Fig. 8:**
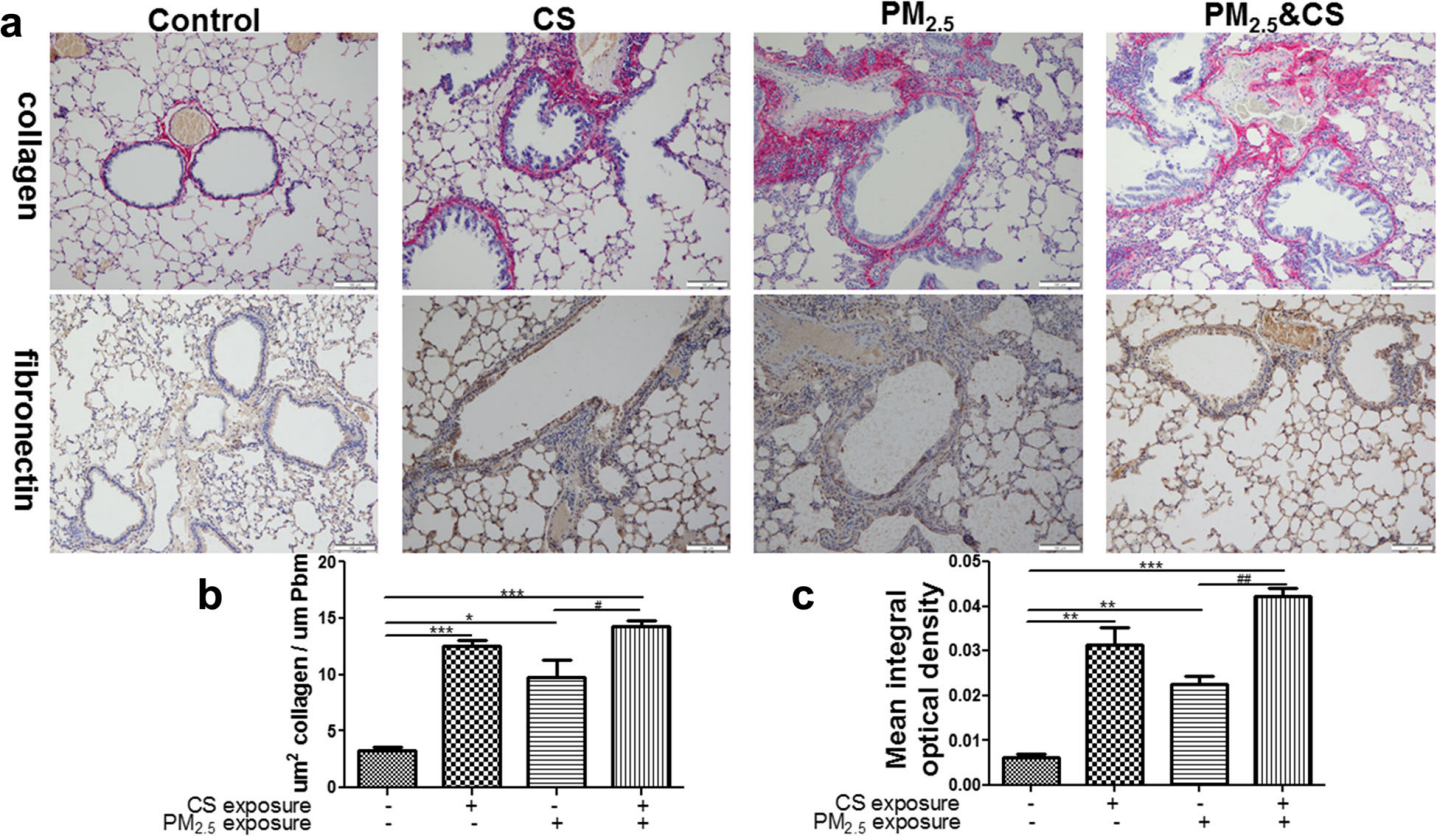
Effect of PM_2.5_ and cigarette smoke exposure on airway wall remodeling. **a** Photomicrographs of peribronchial collagen and fibronectin deposition in lung tissue induced by chronic exposure to PM_2.5_ with or without CS (200X). **b** The assessment of peribronchial collagen deposition in lung tissue induced by chronic exposure to PM_2.5_ with or without CS. **c** Semiquantitative assessment of fibronectin protein expression using Image-Pro Plus. Results were expressed as means± SD (*n* = 6). *P < 0.05, ** *P* < 0.01, *** *P* < 0.001 vs control group; ^#^*P* < 0.05, ^##^ *P* < 0.01, ^###^ *P* < 0.001 vs PM_2.5_ + CSE-exposed group

To further detect the impact of PM_2.5_ exposure on pulmonary extracellular matrix deposition in the airway wall, fibronectin and collagen were evaluated by immunochemistry and Sirius Red, respectively. An evident enhancement in peri-bronchial fibronectin deposition was observed in PM_2.5_/CS group relative to controls (Fig. 8). Similarly, PM_2.5_/CS exposure markedly up-regulated the expression of collagen in peri-bronchial areas. However, fibronectin and collagen protein expression levels in PM_2.5_ + CS group had no clearly significant increase compared to CS group.

## Discussion

COPD is a preventable and treatable pulmonary disease with increasing prevalence worldwide [1]. Numerous epidemiologic studies have revealed the associations between particulate pollution exposure including PM_2.5_ and increased morbidity and mortality of cardiopulmonary diseases, in both developed as well as developing countries [30–32]. However, it remains poorly understood about the underlying effects and associated mechanisms by which PM_2.5_ exposure induces and aggravates COPD development. In this study, we demonstrated that chronic prolonged PM_2.5_ exposure impaired lung function, triggered emphysematous lesions and induced pulmonary inflammation and as well as airway wall remolding. Most importantly, prolonged PM_2.5_ exposure can obviously worsen CS-induced changes of COPD, offering evidence for an important association between PM_2.5_ and cigarette smoking in COPD development and progression.

A 10-μg/m^3^ increase of ambient PM_2.5_ concentration could be related to a 2.5% increase of COPD mortality and 3.1% increment of COPD hospitalizations [33]. However, the extent of relations among different geographic locations varies [31]. In this study, we found that in never-smokers, COPD incidence in the urban cohort was higher than that of the rural cohort. Intriguingly, relative to never-smokers, COPD incidence in the smoking subgroups in the two areas both increased greatly regardless of smoking pack-years. We discovered that PM_2.5_ concentration in the urban was obviously higher than that in the rural. The results demonstrated that the role of PM_2.5_ on COPD incidence in the population was associated with its ambient concentration and smokers were much more susceptible to subject to PM_2.5_ pollution relative to non-smokers. In other words, PM_2.5_ and cigarette smoke could have a synergistic effect on COPD development and progression.

To gain insight into the mechanisms associated between PM_2.5_ and COPD incidence, HBEs were exposed to increased doses of PM_2.5_ with or without CSE to evaluate the levels of pro-inflammatory mediators and important protein markers involved in airway remolding of COPD. Consistent with the results of previous studies, our findings demonstrated that the levels of IL-6 and IL-8 were upregulated in PM_2.5_-exposed cells at a dose-dependent manner [34, 35]. Similarly, PM_2.5_ exposure could also markedly increase MMP9, MMP12, fibronectin, collagen and TGF-β1 protein expression at a concentration-dependent manner. In addition, PM_2.5_ could further strengthen these effects induced by CSE. These results certified that PM_2.5_ not only markedly enhanced the levels of pro-inflammatory cytokines, MMP9, MMP12, fibronectin, collagen and TGF-β1 protein expression, but also reinforced these effects induced by CSE.

To further deeply elucidate the impacts and associated mechanisms of PM_2.5_ on COPD progression and development, other than studies by intra-tracheal or nasal exposure methods [36, 37], we created a chronic prolonged PM_2.5_-exposed animal model for 48 weeks to better simulate the development of COPD over a substantial duration throughout individual life. We discovered that PM_2.5_/CS exposure could markedly induce FEV_0.1_/FVC decline, FEV_0.1_ decrease and FVC increase, indicating that PM_2.5_/CS exposure might lead to lung function decline. Moreover, PM_2.5_ + CS remarkably decreased FEV_0.1_/FVC and FEV_0.1_ relative to their only-counterpart. These results indicated that chronic PM_2.5_ exposure might impair lung function and have a synergistic role with cigarette smoke in triggering much more serious pulmonary function decline.

The persistent airflow limitation characteristic of COPD is caused by chronic inflammation, narrowing of peripheral airways and parenchymal destruction due to emphysema [4]. In the present study, PM_2.5_/CS markedly induced airspace enlargement, alveolar wall destruction and striking pulmonary inflammation. Importantly, we discovered that PM_2.5_ could aggravate CS-related pulmonary emphysematous changes and inflammation.

An imbalance between proteinases and their inhibitors is believed to have a key role in alveolar destruction and airway remolding in COPD development [25, 38]. In addition, TGF-β1 also has an important effect on lung remodeling [39–41]. PM_2.5_/CS clearly enhanced MMP-9 and MMP12 protein expression levels, while there was only a slight increase in the level of TGF-β1. Importantly, an evident enhancement in peri-bronchial fibronectin and collagen deposition were observed in PM_2.5_/CS. Moreover, PM_2.5_ + CS led to an evident enhancement of these expressed protein levels.

## Conclusions

In short, our data suggested that chronic prolonged PM_2.5_ exposure induced impaired lung function, emphysematous lesions, airway inflammation and airway wall remolding. Most importantly, PM_2.5_ exposure could also obviously aggravate CS-induced changes in COPD. This study offers a new insight into the effects induced by chronic PM_2.5_ exposure and associated underlying mechanisms in COPD development and progression. However, it remains unclear whether these changes induced by PM_2.5 _are its direct or indirect exposure effects. Further studies are required to extend these findings.

## Data Availability

Not applicable.

## Abbreviations

BAL: Bronchoalveolar lavage
COPD: Chronic obstructive pulmonary disease
CSE: Cigarette smoke extract
FEV_0.1s_: Forced expiratory volume in 0.1 s
FVC: Forced expiratory vital capacity
HBE: Human bronchial epithelial cells
MLI: Mean linear intercept
MMP12: Matrix Metallopeptidase 12
MMP9: Matrix Metallopeptidase 9
PM_2.5_: Ambient fine particulate matter
TGF-β_1_: Transforming Growth Factor-Beta 1
